# Identification of testis-relevant genes using *in silico *analysis from testis ESTs and cDNA microarray in the black tiger shrimp (*Penaeus monodon)*

**DOI:** 10.1186/1471-2199-11-55

**Published:** 2010-08-09

**Authors:** Thidathip Wongsurawat, Rungnapa Leelatanawit, Natechanok Thamniemdee, Umaporn Uawisetwathana, Nitsara Karoonuthaisiri, Piamsak Menasveta, Sirawut Klinbunga

**Affiliations:** 1 National Center for Genetic Engineering and Biotechnology (BIOTEC), National Science and Technology Development Agency, Klong 1, Klong Luang, Pathumthani 12120, Thailand; 2 Program in Biotechnology, Faculty of Science, Chulalongkorn University, Bangkok 10330, Thailand; 3 Center of Excellence for Marine Biotechnology, Faculty of Science, Chulalongkorn University, Bangkok 10330, Thailand; 4 Department of Marine Science, Faculty of Science, Chulalongkorn University, Bangkok 10330, Thailand

## Abstract

**Background:**

Poor reproductive maturation of the black tiger shrimp (*Penaeus monodon*) in captivity is one of the serious threats to sustainability of the shrimp farming industry. Understanding molecular mechanisms governing reproductive maturation processes requires the fundamental knowledge of integrated expression profiles in gonads of this economically important species. In *P. monodon*, a non-model species for which the genome sequence is not available, expressed sequence tag (EST) and cDNA microarray analyses can help reveal important transcripts relevant to reproduction and facilitate functional characterization of transcripts with important roles in male reproductive development and maturation.

**Results:**

In this study, a conventional testis EST library was exploited to reveal novel transcripts. A total of 4,803 ESTs were unidirectionally sequenced and analyzed *in silico *using a customizable data analysis package, ESTplus. After sequence assembly, 2,702 unique sequences comprised of 424 contigs and 2,278 singletons were identified; of these, 1,133 sequences are homologous to genes with known functions. The sequences were further characterized according to gene ontology categories (41% biological process, 24% molecular function, 35% cellular component). Through comparison with EST libraries of other tissues of *P. monodon*, 1,579 transcripts found only in the testis cDNA library were identified. A total of 621 ESTs have not been identified in penaeid shrimp. Furthermore, cDNA microarray analysis revealed several ESTs homologous to testis-relevant genes were more preferentially expressed in testis than in ovary. Representatives of these transcripts, homologs of *saposin (PmSap) *and *Dmc1 (PmDmc1*), were further characterized by RACE-PCR. The more abundant expression levels in testis than ovary of *PmSap *and *PmDmc1 *were verified by quantitative real-time PCR in juveniles and wild broodstock of *P. monodon*.

**Conclusions:**

Without a genome sequence, a combination of EST analysis and high-throughput cDNA microarray technology can be a useful integrated tool as an initial step towards the identification of transcripts with important biological functions. Identification and expression analysis of *saposin *and *Dmc1 *homologs demonstrate the power of these methods for characterizing functionally important genes in *P. monodon*.

## Background

The black tiger shrimp (*Penaeus monodon*) is an aquatic animal of central importance as it brings an annual income of over one billion USD in Thailand [1]. However, domestication of the black tiger shrimp (*Penaeus monodon*) is impeded by poor reproductive maturation of both male and female brooders in captivity. Ovarian development of penaeid shrimp is induced by a unilateral eyestalk ablation technique; however, the technique does not have the same effect in male reproductive maturation [2]. No molecular markers pinpointing the maturation stage of testis or sperm quality in penaeid shrimp are currently known. Domesticated male *P. monodon *yields lower fertilization rates of zygotes and lower survival rates of offspring than wild male *P. monodon *[3]. However, the role of genes implicated in the regulation of spermatogenesis and their patterns of expression in penaeid shrimp are still poorly understood.

To gain insight on the molecular mechanisms governing the male reproduction process of this important species, EST libraries from *P. monodon *broodstock testis were previously constructed for gene discovery [4,5]. A testis-specific transcript, *PMTST1 *(*P. monodon *testis-specific transcript 1), was identified, and expression levels of 51 additional putative testis-specific genes in cultured and wild *P. monodon *were examined by reverse-transcription (RT)-PCR, semiquantitative RT-PCR, and real-time RT-PCR [5]. Nevertheless, more exhaustive gene discovery is needed to unravel testis-relevant genes and their possible functions.

In this study, a total of 4,803 ESTs from the testis library were sequenced and analyzed *in silico *using a customizable data analysis package, ESTplus [6]. Many transcripts known to be relevant to testicular development in other organisms were identified. A total of 1,076 of these testis EST sequences were included in the construction of a new microarray. Gene expression profiles of testis were simultaneously compared to those of ovary in both juveniles and broodstock. Among transcripts with differential expression levels, *saposin *and *Dmc1 *homologs were further examined by quantitative real-time PCR. Furthermore, full-length sequences of *saposin *and *Dmc1 *cDNAs were obtained by RACE-PCR.

## Results and Discussion

### Characteristics and functional annotation of testis ESTs of *P. monodon*

**Figure 1:**
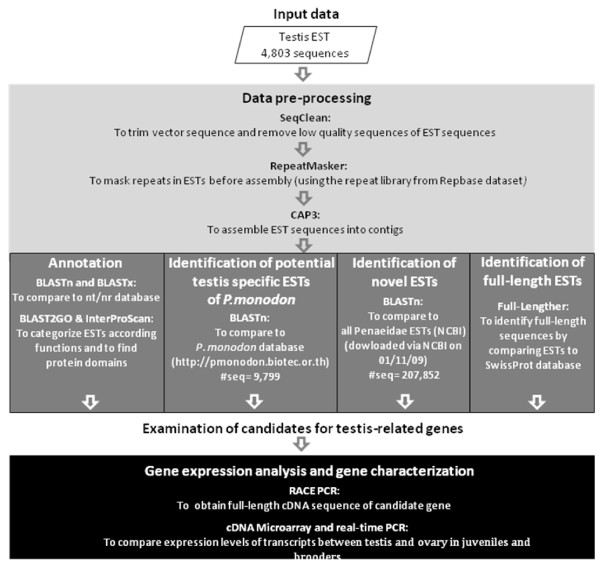
**Overview of data analysis of the testis ESTs in this study**.

**Figure 2:**
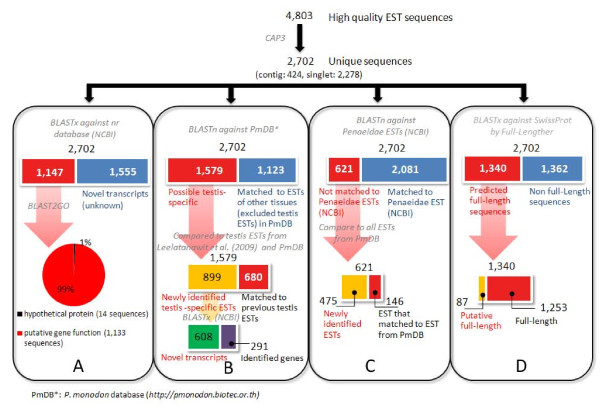
**Results summary of EST data analysis**.

Previously, we constructed suppression subtractive hybridization (SSH) libraries comparing cDNA in testis of wild *P. monodon *broodstock to juveniles. A total of 365 clones were sequenced from these libraries [4]. In addition, we established a high quality conventional cDNA library from testis of wild *P. monodon *broodstock and preliminarily sequenced 896 clones [5]. In this study, additional ESTs were sequenced from the conventional cDNA library and a total of 4,803 high quality EST sequences were obtained (Figure 1A). Results of the *in silico *analysis are summarized in Figure 2. From assembling the testis EST sequences, 2,702 unique sequences comprising of 424 contigs and 2,278 singletons with an average size of 1,130 bp and 584 bp, respectively, were found. When compared with the NCBI nucleotide collection (nr/nt) database using BlastN, 1,181 sequences of the 2,702 unique sequences were found to be homologs of previously reported sequences. Similarly, when the unique EST sequences were compared with the NCBI non-redundant protein sequence database using BlastX, 1,147 (42%) sequences were homologs of previously reported sequences, whereas the remainder (1,555 sequences, 58%) were unknown (novel) transcripts. Matched sequences (1,147) from the BlastX analysis were further categorized by Blast2GO for their potential functions. 1,133 (99%) of these sequences were similar to genes with putative functions (Figure 2A).

**Figure 3:**
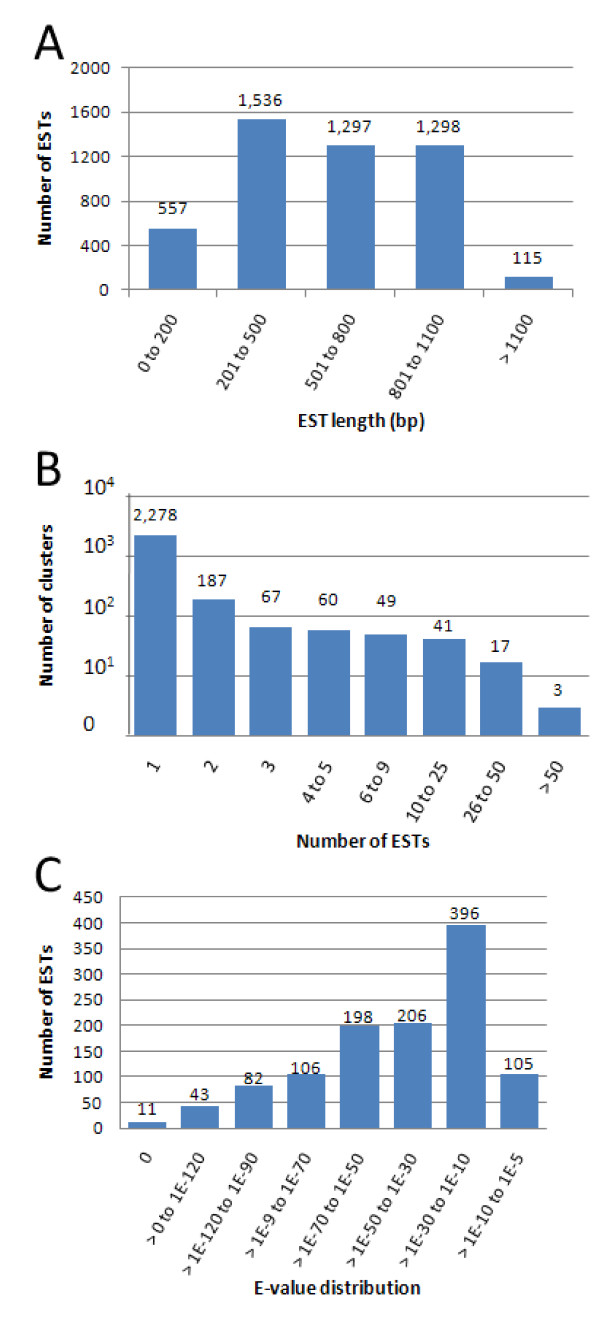
**Distribution of testis ESTs. **(A) Length (in bp) distribution of testis ESTs. (B) Distribution of number of singletons assembled into a contig. (C) Distribution of E-value.

*E*-values of most sequences ranged between 1e-30 to 1e-10 (396 sequences accounting for 34% of overall contigs, Figure 3C). The most frequently matched transcripts found were Arthropoda (753 sequences, 66%) at the phylum level and the red flour beetle *Tribolium castaneum *(90 sequences) at the species level (Table 1). This could be due to the limited availability of *P. monodon *sequence data in comparison to that of *T. castaneum *whose genome was completely sequenced and published in 2008 [7].

**Table 1:** Distribution of species that the testis ESTs were matched to known transcripts in the database using BlastX

**Common taxonomy**	**Phylum**	**Frequency** **(#seq)**	**Percent**	**Species**	**Phylum**	**Frequency** **(#seq)**
	
arthropods	Arthropoda	753	65.6	*Tribolium castaneum*	Arthropoda	90
chordates	Chordata	240	20.9	*Nasonia vitripennis*	Arthropoda	85
plants	Streptophyta	45	3.9	*Apis mellifera*	Arthropoda	75
sea urchins	Echinodermata	32	2.8	*Pediculus humanus*	Arthropoda	66
cnidarians	Cnidaria	29	2.5	*Branchiostoma floridae*	Chordata	65
segmented worms	Annelida	7	0.6	*Ixodes scapularis*	Arthropoda	56
molluscs	Mollusca	5	0.4	*Penaeus monodon*	Arthropoda	55
nematodes	Nematoda	4	0.3	*Triticum aestivum*	Streptophyta	38
placozoans	Placozoa	4	0.3	*Strongylocentrotus purpuratus*	Echinodermata	30
apicomplexans	Apicomplexa	4	0.3	*Acyrthosiphon pisum*	Arthropoda	29
ascomycetes	Ascomycota	2	0.2	*Anopheles gambiae*	Arthropoda	26
ribbon worms	Nemertea	1	0.1	*Aedes aegypti*	Arthropoda	22
trichomonads	Trichomonadida	1	0.1	*Danio rerio*	Chordata	21
kinetoplastids	Kinetoplastida	1	0.1	*Marsupenaeus japonicas*	Arthropoda	21
others		19	1.7	*Nematostella vectensis*	Chordata	18
**Total**		**1,147**		*Litopenaeus vannamei*	Arthropoda	17
				*Xenopus laevis*	Chordata	17
				*Culex quinquefasciatus*	Arthropoda	16
				*Gallus gallus*	Chordata	16
				*Lepeophtheirus salmonis*	Arthropoda	13
				*Salmo salar*	Chordata	12
				*Ornithorhynchus anatinus*	Chordata	11
				*Hydra magnipapillata*	Cnidaria	10
				*others*		338
				**Total**		**1,147**

The 2,702 unique sequences were searched using BlastN against the *P. monodon *EST data (PmDB: http://pmonodon.biotec.or.th) excluding previously identified testis EST libraries (Figure 2B). A total of 1,579 sequences did not match ESTs from other tissues of *P. monodon *making them potential testis-specific transcripts or rare transcripts in other shrimp tissues. Examples of genes in this group relevant to testicular development are *phospholipase A2*, *mago nashi proliferation-associated protein, actin-depolymerizing factor *and *profilin*. *Phospholipase A2 *is required for the acrosome reaction (AR), a special exocytotic process promoted by signal transduction pathways, and capacitation, a process for maturation of spermatozoa [8,9]. Protein mago nashi originally identified in *Drosophila *is essential for germplasm assembly [10]. Actin polymerization is important for a wide range of cellular functions and properties, including cell division, cell motility, cell polarity and cell-cell contacts. Profilins are widely expressed small actin-binding proteins which are functionally involved in the regulation of actin dynamics [11,12]. On the other hand, cofilin/actin-depolymerizing protein is an important factor in spermatogenesis which disassembles actin filaments when unphosphorylated [13].

To reveal novel transcripts which are not found in other *Penaeidae *ESTs, the testis ESTs from this study were also compared to Penaeid ESTs (207,852 sequences, using a keyword of "Penaeidae") from the NCBI database (Figure 2C). A total of 2,081 (77%) sequences were homologous to those from the NCBI's penaeid shrimp database. 621 newly identified transcripts (23%) were found, 475 of which (18%) have not been reported in the PmDB database.

Most of the obtained EST sequences ranged from 201-1,100 bp in length (Figure 3A). Apart from a large number of singletons (2,278 unique sequences, 84% of discovered sequences), most of the contigs (187 contigs, 7%) were assembled from two ESTs and only 100 contigs (4%) were deduced from greater than five ESTs. Only three contigs; *cytochrome C oxidase subunit 1 *(*COI*), *elongation factor 1-alpha *(*EF-1α*), and *cell division cycle 2 *(*cdc2*) were assembled from over 50 sequences (Figure 3B). EF-1α functions in protein synthesis by promoting the binding of aminoacyl tRNA to the 80 S ribosome, and it is one of the most abundant soluble proteins in eukaryotes [14]. Mitochondrial COI has been found to be more highly expressed in well differentiated cells with high activity than in moderately and poorly differentiated cells [15,16]. Cdc2 is the key regulator of the eukaryotic cell cycle, and its activity is controlled by interaction with other proteins such as cyclin A and cyclin B1 [17]. Mitotic cyclins and cdc2 are involved in capacitation and/or acrosome reaction of sperm [18]. During the meiotic cell cycle, the G2/M phase transition is controlled by the maturation promoting factor (MPF), a complex of cdc2 (cdk1) and cyclin B1 [19]. High redundancies of these transcripts in the ESTs are consistent with their essential cellular functions during spermatogenesis and testicular development.

**Figure 4:**
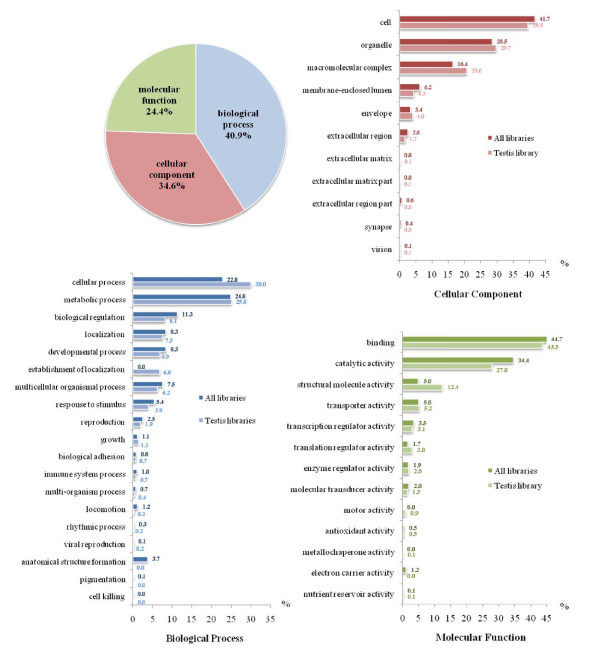
**Functional categories by Gene Ontology (GO) analysis of the testis EST sequences with similarity to the nr database (1,147 sequences total from 2,702 sequences)**. Comparison of different percentages in each GO sub-category between all *P. monodon *ESTs http://pmonodon.biotec.or.th (darker colored bars) and the testis ESTs in this study (lighter colored bars) is represented in all categories.

The assembled EST sequences with similarity to the nr/nt database (1,147 from 2,702 sequences; Figure 2A) were further categorized according to Gene Ontology (Figure 4). The majority of annotated ESTs (41% of 1,147 sequences) were placed in the Gene Ontology functional category of "biological processes". The remaining annotated ESTs were placed in the "cellular components" (35% of 1,147 sequences) and "molecular function" (24% of 1,147 sequences) categories. Compared to the EST sequences from the *P. monodon *EST database, the distribution for the three main categories was similar (42%, 31%, and 27% for biological processes, cellular components and molecular function, respectively).

In the biological process category, ESTs involved in the cellular process were predominant (30% of examined ESTs), followed by those involved in the metabolic process *(*25% of examined ESTs). In the cellular component category, EST functionally involved in the cell (39% of examined ESTs) predominated followed by those functionally displayed in the organelles (30% of examined ESTs) and macromolecular complex (21% of examined ESTs). In the molecular function category, ESTs displaying binding function (43% of examined ESTs) predominated followed by those displaying catalytic activity (28% of examined ESTs).

The reproduction group, including *mago-nashi proliferation-associated protein*, *ubiquitin-conjugating enzyme E2*, and *small ubiquitin-related modifier precursor (SUMO)*, contributes ~2% of the total number of contigs. This discovery rate is comparable to that identified from the conventional ovarian cDNA library of *P. monodon *[20].

The transcripts found in the reproduction group exhibit relevant functions to male reproduction. For instance, SUMO plays an important role in diverse reproductive functions such as spermatogenesis and modulation of steroid receptor activity [21]. In the sumoylation pathway, SUMO is transferred to lysine residues of the protein substrates through the thioester cascade of ubiquitin activating enzyme E1 and ubiquitin conjugating enzyme E2 (UBE2), and SUMO ligase E3 [22]. In the kuruma shrimp *Marsupenaeus japonicus*, UBE2 was expressed at a higher level in testis than in ovary. The expression at stage I (GSI = 0.33 ± 0.004, *N *= 5) was significantly lower than that of stage II (GSI = 0.45 ± 0.12, *N *= 5) but comparable to that of stage III (GSI = 0.57 ± 0.006, *N *= 5) of testis [23]. The analysis of baseline information acquired by this study addresses the paucity of data and provides a better understanding of reproductive maturation in male *P. monodon*.

### Differential Gene Expression Patterns by cDNA Microarrays

**Figure 5:**
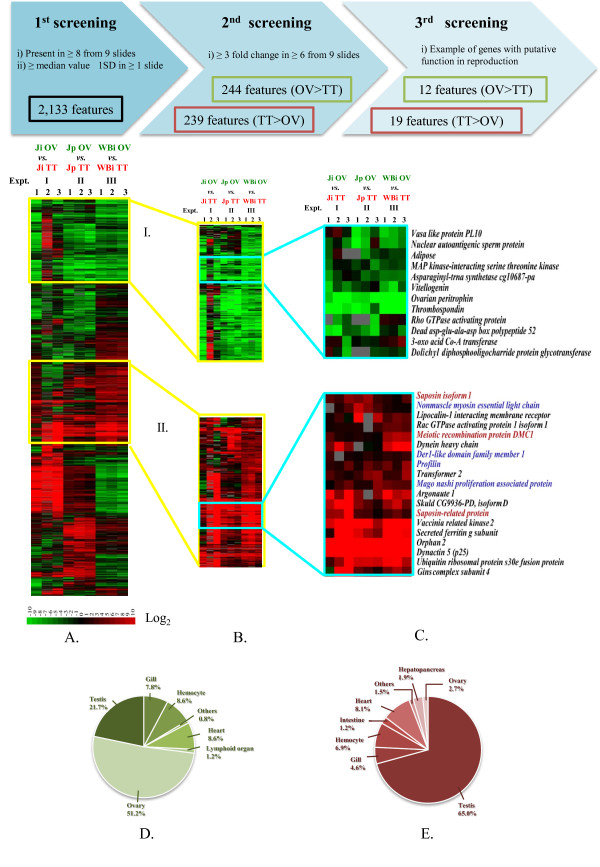
**Gene expression analysis by cDNA microarray comparing *P. monodon *transcripts between testis (TT) and ovary (OV) in individual juvenile (Ji), pooled juvenile (Jp) and individual wild-caught broodstock (WBi)**. TT was labeled with Cy5 fluorescent dye (red) and OV with Cy3 fluorescent dye (green). (A) Hierarchical clustering analysis of the transcripts present in at least 8 of 9 slides and whose expression differences were at least equal to median value ± 1SD in at least 1 slide. (B) Clusters I and II of transcripts with higher expression levels in testis than ovary and *vice versa *with at least 3-fold difference in at least 6 of 9 slides. (C) Examples of differentially expressed transcripts with putative functions in reproductive development. Transcripts in blue letters are those which were not found in any EST libraries of other tissues. *Saposin *and *Dmc1 *(in red) were further characterized by RACE-PCR. (D) Library distributions of transcripts expressed higher in ovary than in testis (244 transcripts) and (E) those expressed higher in testis than in ovary (239 transcripts).

To address the functional involvement of various genes during reproductive development and maturation in *P. monodon*, we previously constructed a cDNA microarray, *Repro*Array^GTS^, and carried out a high-throughput expression analysis of genes in ovary of wild *P. monodon *[24]. A second generation cDNA microarray was recently fabricated using 12 EST libraries from different tissues to cover as many and as diverse transcripts as possible. Expression levels of EST transcripts with gene ontology related to reproductive functions and those previously not reported in the NCBI EST penaeid shrimp database (unique genes) were investigated by using this new microarray to compare ovary and testis transcripts in both juvenile and broodstock shrimp (Figure 5). The expression levels from the microarray experiments were analyzed, as described in the Methods section, to obtain transcripts potentially relevant to testicular development.

Of 5,568 transcripts on the microarray, 2,133 transcripts were present in at least 8 of the 9 microarrays and had relative expression levels greater than the median value ± 1SD in at least one microarray (Figure 5A). Within this subset, 244 transcripts with higher abundance (3 fold) in ovary than testis and 239 transcripts with higher abundance (3 fold) in testis than ovary were identified (Figure 5B). From these differentially expressed genes, there were transcripts whose putative functions are possibly involved in testicular development (Figure 5C).

When examining the library sources of transcripts more abundantly expressed in ovaries, we found 51% were from the ovary, 22% from testis, 9% from hemocyte and heart each, 8% from gill, 1% from lymphoid organ and <1% from the remaining tissues (Figure 5D). These transcripts included *peritrophin *(also called *cortical rod protein*, *CRP*), *thrombospondin *(*TSP*) and *nuclear autoantigenic sperm protein *(*NASP*). In the kuruma shrimp, *CRP *and *TSP *are first scattered throughout the cytoplasm of oocytes and are subsequently localized in cortical rods during the cortical rod formation [25]. NASP is a histone-binding protein. Overexpression of NASP in mice testis affects progression of cell proliferation through the cell cycle [26,27]. Expression analysis of these genes [20,28] and *dolichyl diphosphooligocharide protein glycotransferase*, *asparagenyl tRNA synthetase *and *3-oxoacid CoA transferase *by semiquantitative RT-PCR (S. Klinbunga, unpublished data) revealed greater expression levels in ovary than testis of wild *P. monodon *broodstock. This validated the accuracy of the microarray analysis for gene expression analysis in *P. monodon*.

Transcripts with higher expression in testis were found mainly from the testis EST library (65%), 8% from heart, 7% from hemocyte, 5% from gill, 3% from ovary, 2% from hepatopancreas, 1% from intestine, and 1.5% from the rest (Figure 5E). Examples of these transcripts include *argonaute 1*, *dynactin 5*, and *orphan 2*. Argonaute proteins are key mediators for RNA silencing mechanisms [29]. Cytoplasmic dynactin forms a complex with dynein and plays a functional role in spermatid growth in *Drosophila *[30].

Based on the distribution of library sources where the differentially expressed transcripts came from, it is evident that we can obtain additional information by including cDNA probes from various EST libraries beyond testis and ovary libraries. This more comprehensive coverage of microarray probes demonstrates the usefulness of the present microarray version in gene expression analysis of *P. monodon*.

Within the 1,076 cDNA spots from testis ESTs that were fabricated onto the present version of cDNA microarrays, there were 169 transcripts with greater abundance in testis than ovary. Of these transcripts *saposin-related protein *and *Dmc1 *were consistently more abundant in testis than in ovary for both juvenile and broodstock comparisons. Moreover, these genes have previously been reported to be relevant to male reproduction in other organisms.

In vertebrates, saposins are a group of four small glycoproteins (A, B, C, and D), derived from a common precursor protein called prosaposin, which is reported to activate glycosphingolipid hydrolysis [31]. Inactivation of prosaposin gene resulted in accumulation of lactosylceramide, glucosylceramid, digalactosyl ceramide, sulfactide, ceramide, and globotriaosylceramide in lysosomes. A prosaposin -/- mouse model demonstrated that the mice die at day 35-40 after birth due to neurological defects [32]. The disruption of the prosaposin gene resulted in a reduction in size and weight: 30% of the testis, 37% of the epididymis, 75% of the seminal vesicles, and 60% of the prostate glands. Moreover, the smaller testes from the mutant mice were associated with reduced spermiogenesis, especially in the late spermatids [33].

Dmc1 (a RAC A-like recombinase) is known to be a specific factor for meiotic recombination and has been identified as a gene product specifically expressed during the early meiotic prophase [21]. Recently, the full-length *Dmc1 *cDNA was cloned from the testis of the Japanese eel (*Anguilla japonica*). *Dmc1 *mRNA was abundantly expressed in the testis and ovary with lower expressed in the brain. *In situ *hybridization revealed that *Dmc1 *of *A. japonica *was localized only in the primary spermatocytes implying its important role during the initial stages of spermatogenesis [34].

### Isolation and characterization of the full-length cDNA of *saposin *(*PmSap*) and *Dmc1 *(*PmDmc1*) in *P. monodon*

Based on their differential expression levels seen in the microarray analysis and their possible involvement in testicular development from previous studies in other organisms, *saposin-related protein (PmSap) *and *Dmc1 (PmDmc1) *were further characterized by RACE-PCR to obtain their full-length cDNA sequences.

**Figure 6:**
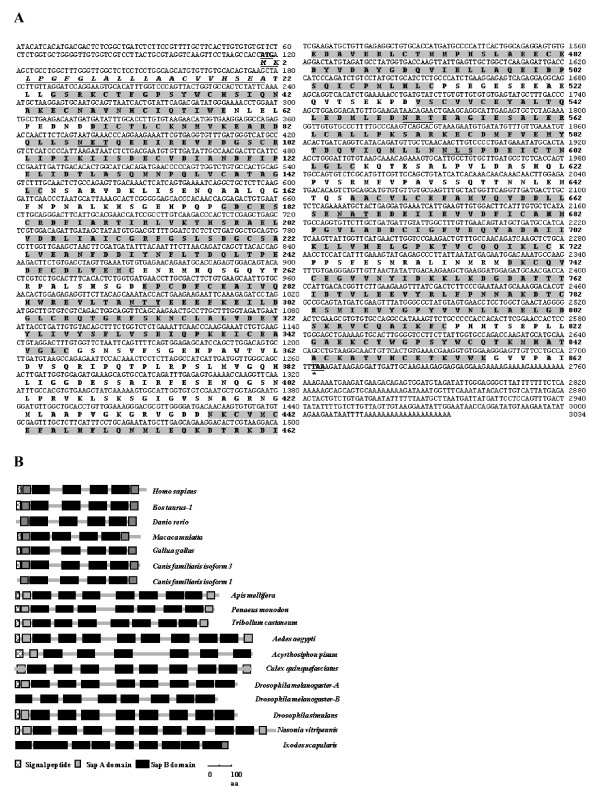
**Analysis of *Penaeus monodon Saposin (PmSap)*.** (A) The full-length cDNA and deduced protein sequence of *PmSap *(3034 bp; ORF 2589 bp, 862 aa) of *P. monodon*. Start (ATG) and stop (TAA) codons are illustrated in boldface and underlined. The signal peptide of the deduced PmSap protein is italicized and underlined. Putative *N*-linked glycosylation sites are underlined. Two domains of Saposin A (positions 25-58 and 823-856) and seven domains Saposin B (positions 68-144, 178-251, 272-346, 437-512, 531-606, 646-721, and 738-813 of the deduced protein) are highlighted. (B) Diagram showing SapA and SapB domains in prosaposin in various vertebrates; *Homo sapiens *(CAI40836), *Bos taurus *(NP_776586), *Macaca mulatta *(XP_001106592), *Canis familiaris *(XP_536382 and XP_861441), *Gallus gallus *(NP_990142), *Danio rerio *(AAH63994) and saposin-related protein in invertebrates; *Acyrthosiphon pisum *(XP_001943244), *Apis mellifera *(XP_392338), *Tribolium castaneum *(XP_966852), *Aedes aegypti *(XP_001662994), *Culex quinquefasciatus *(XP_001864689), *Drosophila melanogaster *(NP_524597 and NP_733408), *Drosophila simulans *(XP_002105562), *Nasonia vitripennis *(XP_001603446) and *Ixodes scapularis *(XP_002412058). Sequenced were retrieved from the GenBank and compared with saposin-related protein of *P. monodon*. Four SapB domains are conserved in vertebrate saposins whereas the number of SapB domains varies among invertebrate taxa.

Full-length *PmSap *was found to be 3,034 bp in length containing an open reading frame (ORF) of 2,589 bp corresponding to a deduced protein of 862 amino acids with 5' and 3'UTRs of 116 and 329 bp (excluding the poly A tail), respectively (Figure 6A, accession number GU566728). This sequence significantly matched *saposin isoform 1 *of *Tribolium castaneum *(*E*-value = 1e-171). The expected molecular weight (MW) and p*I *of the deduced Saposin were 95.63 kDa and 4.65, respectively. Only one contig of *saposin-related protein *was previously found in ESTs from *P. monodon*.

Most saposins in vertebrate and invertebrate contain a signal peptide. Typically, vertebrate prosaposins contain two conserved SapA domains and four conserved SapB domains in which six equally placed cysteines are found in each SapB domain. In contrast, the numbers of saposin domains in invertebrates is less conserved. The absence of some of SapA domain produced variants of saposin-related proteins in various taxa (Figure 6B). Moreover, there are variable numbers of SapB domains in invertebrates, from 6 domains present in the pea aphid, *Acyrthosiphon pisum *(Homoptera), to up to 9 domains in the black legged tick *Ixodes scapularis *(Acari) and *Nasonia vitripennis *(Hymenoptera) (Figure 6B). Additional SapB domains in invertebrate saposins may be explained by several rounds of tandem internal gene duplication as previously proposed for the creation of the four domain saposins in vertebrate [35].

In *P. monodon*, the deduced PmSap protein contained a signal peptide with a cleavage site between Ala_21 _and Glu_22_, two SapA and seven SapB domains (*Sap*A: positions 25-58 and 823-856 with *E*-value = 2.74e-12 and 1.56e-07, respectively; *Sap*B: positions 68-144, 178-251, 272-346, 437-512, 531-606, 646-721, and 738-813 with *E*-value = 1.32e-22, 5.32e-09, 7.28e-16, 4.34e-23, 4.61e-27, 2.63e-22, and 5.83e-15, respectively). Interestingly, six fixed positions of cysteine were found in each SapB domain of PmSap but conserved prolines in identical positions as previously reported in vertebrate saposins [31] were not found (data not shown). Five predicted glycosylation sites were found in SapB domains 1 (NET, positions 87-89), 3 (NTT, positions 291-293), 5 (NRT and NLS, positions 550-552 and 593-595, respectively) and 6 (NAT, positions 665-667). These predicted SapB domains significantly matched saposin-related protein A (*E*-value = 8e-24; Uniprot No. Q9Y125), BmP109 (*E*-value = 7.4e-20; Uniprot No. O15997), Saposin C (*E*-value = 6.4e-14; Uniprot No. P220097) and BmP109 (*E*-value = 3.4e-7; Uniprot No. O15997), respectively.

**Figure 7:**
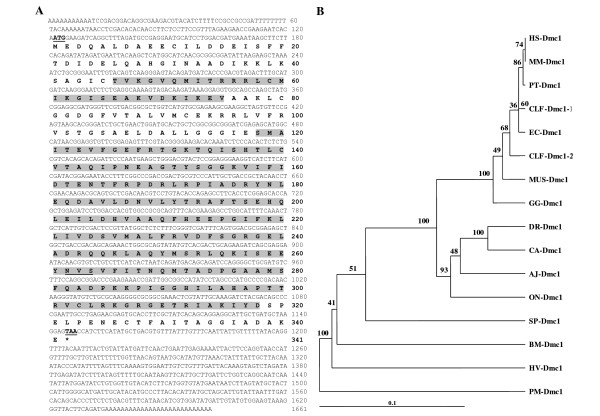
**Analysis of *Penaeus monodon Dmc1 (PmDmc1)*. (**A) The full-length cDNA and deduced protein sequences of *Dmc1 *(1,661 bp; ORF 1,026 bp, 341aa) of *P. monodon*. Start (ATG) and stop (TAA) codons are illustrated in boldface and underlined. The deduced DMC1 protein contains an HhH domain (positions 46-75, *E-*value = 2.7e-02; highlighted)) and an AAA domain (positions 118-308, *E*-value = 9.73e-06; highlighted). (B) A bootstrapped neighbor-joining tree illustrating phylogentic relationships of *Dmc1 *of various taxa. Values at the node represent the percentage of times that the particular node occurred in 1000 trees generated by bootstrapping the original aligned protein sequences. Protein sequences of different isoforms of *Dmc1 *from *Homo sapiens *(*HS-Dmc1*, NM_007068), *Equus caballus *(*EC-Dmc1*, XM_001501584), *Macaca mulatta *(*MM-Dmc1*, XM_001094012), *Pan troglodytes *(*PT-Dmc1*, XM_515130), *Canis lupus familiaris *(*CLF-Dmc1-1*, XM_844891 and *CLF-Dmc1-2*, XM_855217), *Gallus gallus *(*GG-Dmc1*, XM_425477), *Mus musculus *(*MM-Dmc1*, NM_010059), *Anguilla japonica *(*AJ-Dmc1*, AB182645), *Oreochromis niloticus *(*ON-Dmc1*, AB182646), *Danio rerio *(*DR-Dmc1*, NM_001020782), *Carassius auratus *(*CA-Dmc1*, EF545136), *Bombyx mori *(*BM-Dmc1*, NM_001044087), *Hydra vulgaris *(*HV-Dmc1*, AB047581) and *Strongylocentrotus purpuratus *(*SP-Dmc1*, XM_786187), were retrieved from GenBank and compared with *Dmc1 *of *P. monodon *(*PmDmc1*).

The *Dmc1 *transcript which was only found in the testis cDNA library of *P. monodon *was further characterized. The full-length cDNA of *PmDmc1 *(accession number EU440762) was found to be 1,661 bp in length containing an ORF of 1,026 bp encoding a polypeptide of 341 amino acids. The highest similarity to this transcript was *Dmc1 *of *Ixodes scapularis *(*E*-value = 1e-147). The predicted p*I *and MW of the deduced Dmc1 were 5.35 and 37.54 kDa, respectively. The deduced Dmc1 protein contains an HhH motif (positions 46 - 75, *E*-value = 2.73e-02) and an AAA domain (positions 118 -308, *E*-value = 9.73e-06, Figure 7A). The HhH motif is ~20 amino acid domain present in prokaryotic and eukaryotic non-sequence-specific DNA binding proteins involved in enzymatic activity. The gene products within the superfamily of AAA-ATPase are associated with diverse cellular activity. A bootstrapped neighbor-joining tree reflects the relationships between the texa and the sparse representation of invertebrate sequence for the Dmc1 protein (Figure 7B).

### Expression of *PmSap *and *PmDmc1 *in testis of juveniles and domesticated and wild broodstock of *P. monodon*

Tissue expression analysis is important for providing basic information needed to prioritize further analysis of functional genes. Based on the fact that a particular gene may be expressed in several tissues and possess different functions, rapid detection of *PmSap *and *PmDmc1 *expression profiles in gonads of *P. monodon *by cDNA microarray was further confirmed by quantitative real-time PCR.

**Figure 8:**
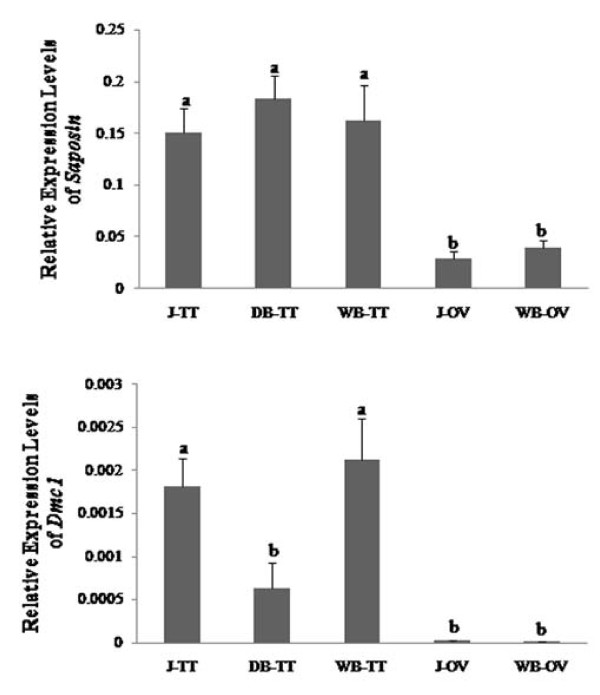
**Real-time PCR analysis illustrating the relative expression levels of *saposin *in testis (TT) and ovary (OV) of *P. monodon *juvenile (J), domesticated (DB) and wild broodstock (WB)**. Bars labeled with the same letters do not have significantly different expression levels (*P *> 0.05). J-TT (*N *= 5), DB-TT (*N *= 5), and WB-TT (*N *= 5) are testis of juveniles, domesticated broodstock, and wild broodstock, respectively. J-OV (*N *= 4) and WB-OV (*N *= 4) are ovary of juvenile and wild broodstock, respectively.

*PmSap *was more abundantly expressed in testis than ovary of *P. monodon *(*P *< 0.05, Figure 8). The expression levels of this transcript were not significantly different across different stages of either testis (WB-TT, DB- TT and J-TT) or ovary (WB- TT and J-TT). *Dmc1 *was less abundantly expressed in testis of domesticated broodstock than in wild broodstock and cultivated juvenile *P. monodon *males (*P *< 0.05, Figure 8). The expression level of this gene was also higher in testis than ovary (*P *< 0.05). Therefore, *PmSap *and *PmDmc1 *should play an important role in spermatogenesis (and testicular development) rather than oogenesis (and ovarian development) of *P. monodon*. Importantly, lower expression levels of *PmDmc1 *in domesticated versus wild broodstock suggest transcriptional levels of this gene may be used to indicate possible reduced maturation of reproduction in domesticated *P. monodon*.

Molecular mechanisms and expression patterns of genes controlling different steps of sperm maturation and testicular development should be examined for a better understanding of *P. monodon *reproductive maturation in captivity. The limited number of known genes expressed in testis of this economically important species was partially resolved by EST analysis in the present study. Typically, molecular studies of the hormonal and environmental effectors involving shrimp reproduction have been limited to one or a few genes. Here, we illustrate that cDNA microarrays are relatively simple, reliable and cost-effective for examining integrated interactions among genes in gonads of *P. monodon*. This method can help accelerate studies on reproductive development and maturation of *P. monodon*.

## Methods

### EST Libraries

The testis cDNA library of *P. monodon *was previously constructed as described in [5]. The lambda library was converted to the pBluescript library by *in vivo *excision. Colony PCR was performed in a 25 μl reaction mixture containing 10 mM Tris-HCl, pH 8.3, 50 mM KCl, Enhancer solution, 200 mM of each dNTP, 2 mM MgCl_2_, 0.2 μM each of M13-F (5'-ACG TTG TAA AAC GAC GGC CAG-3') and M13-R (5'-ACA GGA AAC AGC TAT GAC CAT G-3'), and 1.25 unit of *i-Taq*™DNA Polymerase (iNtRON Biotechnology). PCR was carried out in a thermocycler consisting of predenaturation at 94°C for 5 min followed by 35 cycles of denaturation at 94°C for 30 s, annealing at 58°C for 1 min and extension at 72°C for 3 min. The final extension was carried out at the same temperature for 7 min. The colony PCR products were size-fractionated on 1.5% agarose gel and visualized after ethidium bromide staining. Clones with >400 bp inserts were selected for further sequencing. Plasmid was extracted using 96-well plate format (EZ-10 96-well spin column, Bio Basic). The concentrations of plasmids were measured using a NanoDrop 8000. Nucleotide sequence of each clone was obtained using an automated DNA sequencer. The 4,803 sequences of the testis ESTs from this study were deposited in NCBI dbEST http://www.ncbi.nlm.nih.gov/dbEST/ with accession numbers of GW992816-GW996323, HO000142-HO000184, GE613296 - GE614547.

### *In silico *analysis of ESTs

Nucleotide sequences were analyzed using ESTplus, an integrative system for comprehensive and customized EST analysis and proteomic data matching [6], as summarized in Figure 1. Briefly, the sequencing data were analyzed by SeqClean (removal of the polyA/polyT tail, low-quality ends, short sequences and those containing cloning vector), RepeatMasker (masking of the sequences when matched with sequences in the largest repeat library, the RepBase, that covers a number of organisms including human, rodent, zebrafish, *Drosophila*, and *Arabidopsis thaliana*) (University of Washington Genome Center, Seattle; http://ftp.genome.washington.edu/cgi-bin/RepeatMasker), and CAP3 (assembly and clustering of EST sequences) [36]. The post-processed sequences were further annotated for biological activities by comparison with the NCBI nr database using BlastN (a nucleotide-level annotation, *E*-value < 10^-5^), BlastX (a protein-level annotation, *E*-value < 10^-5^), BLAST2GO (Gene Ontology prediction of the annotated proteins from BlastX program), and InterProScan (protein signature identification).

To identify ESTs found only in the testis cDNA library of *P. monodon*, all newly sequenced contigs and singletons were compared against all sequences (38,429 ESTs) from the *P. monodon *database (PmDB: http://pmonodon.biotec.or.th). Moreover, to identify novel transcripts that have not been previously reported in shrimp, the sequenced data were also compared against all available Penaeidae ESTs (207,852 ESTs) from NCBI http://ncbi.nlm.nih.gov retrieved on 01/11/09.

### Microarray analysis for identification of differential expression transcripts in testis of *P. monodon*

A cDNA microarray was constructed from various EST libraries of *P. monodon*, consisting of 5,568 features (1,076 unique cDNA features were amplified from testis EST libraries). The arrays were post-processed according to [24] immediately before hybridization.

Total RNA was extracted from testis and ovary of juveniles (4 month-old) and broodstock originating from the Andaman Sea (west of peninsular Thailand). Contaminating genomic DNA was removed by treatment with DNase I (0.15 U/μg total RNA) at 37°C for 30 min. The first-strand cDNA was synthesized and fluorescently labeled according to [24]. RNA from ovary was labeled with Cy3 dye as a reference and RNA from testis was labeled with Cy5 dye. The Cy3- and Cy5-samples were mixed together and hybridized onto the arrays overnight. The hybridized slides were washed according to Corning's instruction. Nine microarray experiments were performed and the details of comparison between gene expression profiles of ovaries and testes are summarized in Table 2.

**Table 2:** Summary of microarray experiments in this study

Experiment	Female Green (Cy3)	Male Red (Cy5)
*I. Comparison of gene expression levels between ovary and testis in individual juveniles*		
1	Ovary from juvenile 1	Testis from juvenile 1
2	Ovary from juvenile 2	Testis from juvenile 2
3	Ovary from juvenile 3	Testis from juvenile 3
*II. Comparison of gene expression levels between ovaries and testes pooled from juveniles*		
1	Pooled ovaries from juveniles (n = 98)	Pooled testes from juveniles (n = 114)
2	Pooled ovaries from juveniles (n = 98)	Pooled testes from juveniles (n = 114)
3	Pooled ovaries from juveniles (n = 98)	Pooled testes from juveniles (n = 114)
*III. Comparison of gene expression levels between ovaries and testes pooled from broodstock*		
1	Ovary from broodstock 1 (GSI*: 12.4)	Testis from broodstock 1 (GSI: 1.1)
2	Ovary from broodstock 2 (GSI: 11.2)	Testis from broodstock 2 (GSI: 1.1)
3	Ovary from broodstock 3 (GSI: 12.6)	Testis from broodstock 3 (GSI: 1.1)

*Gonadosomatic Index (GSI) = % gonad weight/body weight

### Microarray imaging and data analysis

The hybridized slides were scanned with a GenePix 4000B (Molecular Devices, Sunnyvale, CA) and processed using GenePix Pro version 6.1. Only spots with intensities greater than one standard deviation above the background intensity were further analyzed. The processed data were normalized within each array by the scaled print-tip (Lowess) method, and across arrays, using the Aroma package [37], (available from http://www.maths.lth.se/help/R/aroma/, run in R project environment http://cran.r-project.org. The microarray data have been deposited in NCBIs Gene Expression Omnibus http://www.ncbi.nlm.nih.gov/geo/ with GEO accession number GSE19037 at [38]. The average logarithmic base 2 values of relative intensities between Cy3- and Cy5- samples (M values) were subjected to hierarchical clustering analysis and illustrated using the Treeview software [39]. To identify differential expressed transcripts, only transcripts with relative expression level changes more than the median value ± 1SD in at least 1 array and present in at least 8 of the 9 arrays were further considered. A 3-fold change in at least 6 of the 9 arrays was considered as criteria for differential expression.

### Examination of full-length cDNA sequences of *Saposin *and *Dmc1 *by Rapid Amplification of cDNA Ends-Polymerase Chain Reaction (RACE-PCR)

Gene-specific primers for RACE-PCR of *P. monodon Saposin *and *Dmc1 *were designed (Table 3). RACE-PCRs of *Saposin *and *Dmc1 *were carried out using a BD SMART RACE cDNA Amplification Kit following the protocol recommended by the manufacturer (BD Biosciences Clontech). The amplified fragment of each gene was electrophoretically analyzed and eluted from an agarose gel before cloning into pGEM-T Easy vector (Promega) and sequencing [40]. The full-length cDNA was assembled from EST and RACE-PCR products (hereafter called *PmSap *and *PmDmc1*). The sequences of *PmSap *and *PmDmc1 *were deposited with accession numbers of GU566728 and EU440762, respectively. The functional domains of the deduced amino acids of these genes were analyzed using SMART http://smart.embl-heidelberg.de/. The p*I *and molecular weight were estimated using ProtParam http://www.expasy.org/tools/protparam.html.

**Table 3:** Primer sequence, melting temperature and the expected amplification product of *PmSap *and *PmDmc1*

Gene/Primer	Sequence	Tm (°C)	Size (bp)
**RACE-PCR**			
***Saposin***			
5'-RACE-1	5'-CTGGCACCAATAAGAAGGACCCCAAGTG-3'	86	-
5'-RACE-2	5'-CAGATGGGCAGAGATGCAGCATAGGACA-3'	86	-
***Dmc1***			
5'-RACE	5'-GCCGCCAATCGGTTTCTTCGGGTCC-3'	82	-
3'-RACE	5'-CCTTCGCTATCACAGCAGGAGGCATTG-3'	84	-

**Real-time PCR**			
***Saposin***			
Standard curve	F: 5'-GCTATGGTTCAGGTTGATGACTTGC-3'	74	614
	R: 5'-ACTCCCTTCCACACCTTCGTTTC-3'	70	
Real-time PCR	F: 5'-CCATAAAGTTCTGCCCCCACCAC-3'	68	145
	R: 5'-CCCTTCCACACCTTCGTTTCACA-3'	70	
***EF-1α***			
Standard curve	F: 5'-GCTCTTACCGAGGCTGTCCC-3'	66	434
	R: 5'-GTGGGTGTAATCAAGGAGGTCAA-3'	68	
Real-time PCR	F: 5'-TTCCGACTCCAAGAACGACC-3'	62	122
	R: 5'-GAGCAGTGTGGCAATCAAGC-3'	62	
***Dmc1***			
Standard curve	F: 5'-ATGGAAGATCAGGCTTTAGATGC-3'	66	425
	R: 5'-GTGACGCAGAGAGTGTGGGAG-3'	68	
Real-time PCR	F: 5'-ATGTGCGAGAAGCGAAGGC-3'	60	150
	R: 5'-GCAGAGAGTGTGGGAGATTTGTG-3'	70	

### Gene expression analysis of *PmSap *and *PmDmc1 *by quantitative real-time PCR

Gene-specific primers were designed for *PmSap*, *PmDmc1 *and *Elongation factor 1 alpha *(*EF-1α*, Table 3). For construction of the standard curve of each transcript, a plasmid containing the transcript was constructed by cloning PCR product into pGEM-Teasy vector and transforming the resulting vector into *E. coli *JM109. The plasmid was extracted and used as the template for construction of the standard curve by 10-fold serial dilutions (10^3 ^- 10^9 ^copy numbers). Real-time PCR reactions were carried out in a 96-well plate in triplicate.

The expression levels of *PmSap *and *PmDmc1 *in testis from juvenile (J-TT, *N *= 5), domesticated broodstock (DB-TT, *N *= 5), and wild broodstock (WB-TT, *N *= 5) and ovary from juvenile (J-OV, *N *= 4) and wild broodstock (WB-OV, *N *= 4) were further analyzed by quantitative real-time PCR analysis. *EF-1α *was used as an internal control. Each primer set was designed to generate 120 to 150 bp amplicons (Table 3). Each reaction was performed in a 15 μl reaction volume containing 2× LightCycler 480 SYBR Green I Master (Roche), 50 ng of first strand cDNA template, and 0.2 μM or 0.4 μM of each primer pair. Cycling parameters were 95°C for 10 min, 40 cycles of 95°C for 15 sec, 58°C for 30 sec, and 72°C for 30 sec. The specificity of PCR products was confirmed by melting curve analysis by heating at 95°C for 15 sec, 65°C for 1 min before heating to 97°C and gradually cooling down to 40°C within 10 sec. Real-time PCR of each shrimp was tested in duplicate. Relative expression levels (copy number of the target gene/that of *EF-1α*) of different sample groups were statistically tested by ANOVA followed by Duncan's new multiple range test or Tukey test (*P *< 0.05).

## Authors' contributions

RL and SK constructed the testes EST library. TW, RL and NT carried out plasmid isolation and sequencing. TW performed *in silico *analysis of cDNA sequences. UU, TW and NK constructed cDNA microarray chips and performed microarray experiments. RL carried out gene expression analysis experiments and full-length characterization. PM, NK and SK conceived the work. TW, RL, NK and SK wrote the manuscript. All authors read and approved this manuscript.
